# Supplementary material for: Transport and Utilization of Glycogen Breakdown Products by Gardnerella spp. from the Human Vaginal Microbiome
Source: Microbiol Spectr. 2023 Mar 15;11(2):e04435-22. doi: 10.1128/spectrum.04435-22 (PMC10101108; doi:10.1128/spectrum.04435-22)
Supplement: Supplemental file 1 — Table S1 and Fig. S1 and S2. Download spectrum.04435-22-s0001.pdf, PDF file, 1.6 MB [file spectrum.04435-22-s0001.pdf]

Supplemental Table 1: Summary of the total number of bases, read lengths and coverage produced by Nanopore whole genome sequencing

Species	Isolate	Total number of contigs	Total length of assembly (bp)	Min. read length (bp)	Max read length (bp)	Average read length (bp)	Coverage (×)	Busco score	Circular contig (Y/N*)
G. leopoldii	GH005	1	708,331,962	1,000	70,429	3,749	482	87.3	Y
	NR017	1	625,880,717	1,000	89,494	6,144	412	84.5	Y
	VN003	1	50,269,029	1,000	95,806	6,583	34	87.3	Y
G. piotii	GH007	2	447,961,852	1,000	109,288	4,104	293	84.5	Y
	GH020	2	347,965,445	1,000	75,228	4,785	227	85.6	Y
	VN002	1	391,732,907	1,000	69,940	3,041	254	84.2	Y
G. swidsinskii	NR016	1	933,619,903	1,000	78,019	3,327	569	84.5	Y
	NR020	1	542,292,693	1,000	96,569	6,804	339	84.2	Y
	NR021	1	704,597,152	1,000	58,607	3,313	458	79.8	Y
G. vaginalis	NR001	1	419,092,692	1,000	75,848	7,069	259	79.5	Y
	NR038	1	241,732,137	1,000	77,337	7,243	145	85.6	Y
	NR039	1	356,241,041	1,000	89,707	7,181	216	85.6	Y
Genome sp. 3	N170	1	404,975,009	1,000	106,723	8,592	265	84.9	Y
	NR026	1	260,150,009	1,001	67,489	8,126	164	85.2	Y
	NR047	2	408,699,904	1,000	68,142	6,475	269	85.6	Y

(* Y: Yes, N: No)

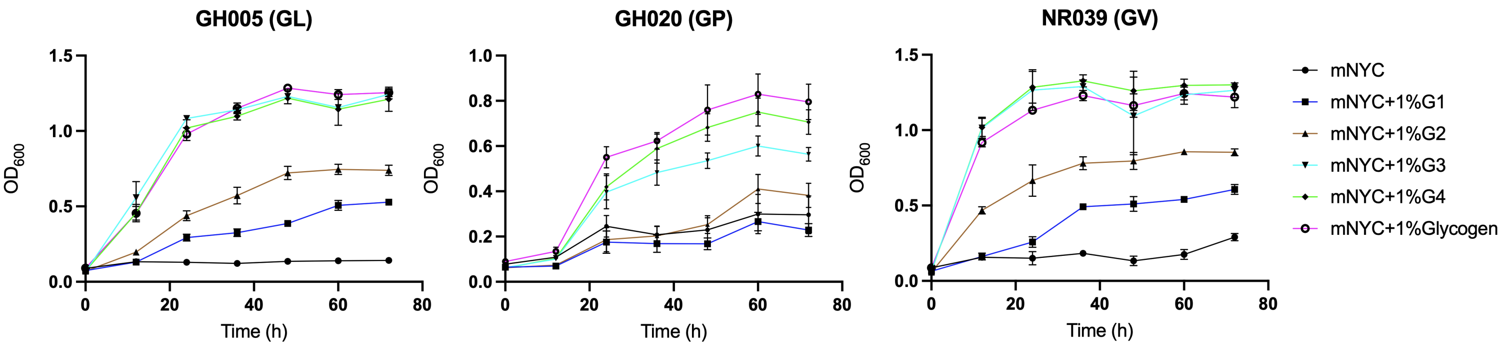

Supplemental Figure 1: Growth curves of 3 representative *Gardnerella* isolates (GL: G. leopoldii, GP: G. piotii and GV: G. vaginalis) in mNYC media, mNYC supplemented with 1% glucose, 1% maltose, 1% maltotriose, 1% maltotetraose or 1% glycogen. All data are an average of two biological replicates each with three technical replicates. Error bars represent standard deviation above and below the mean.

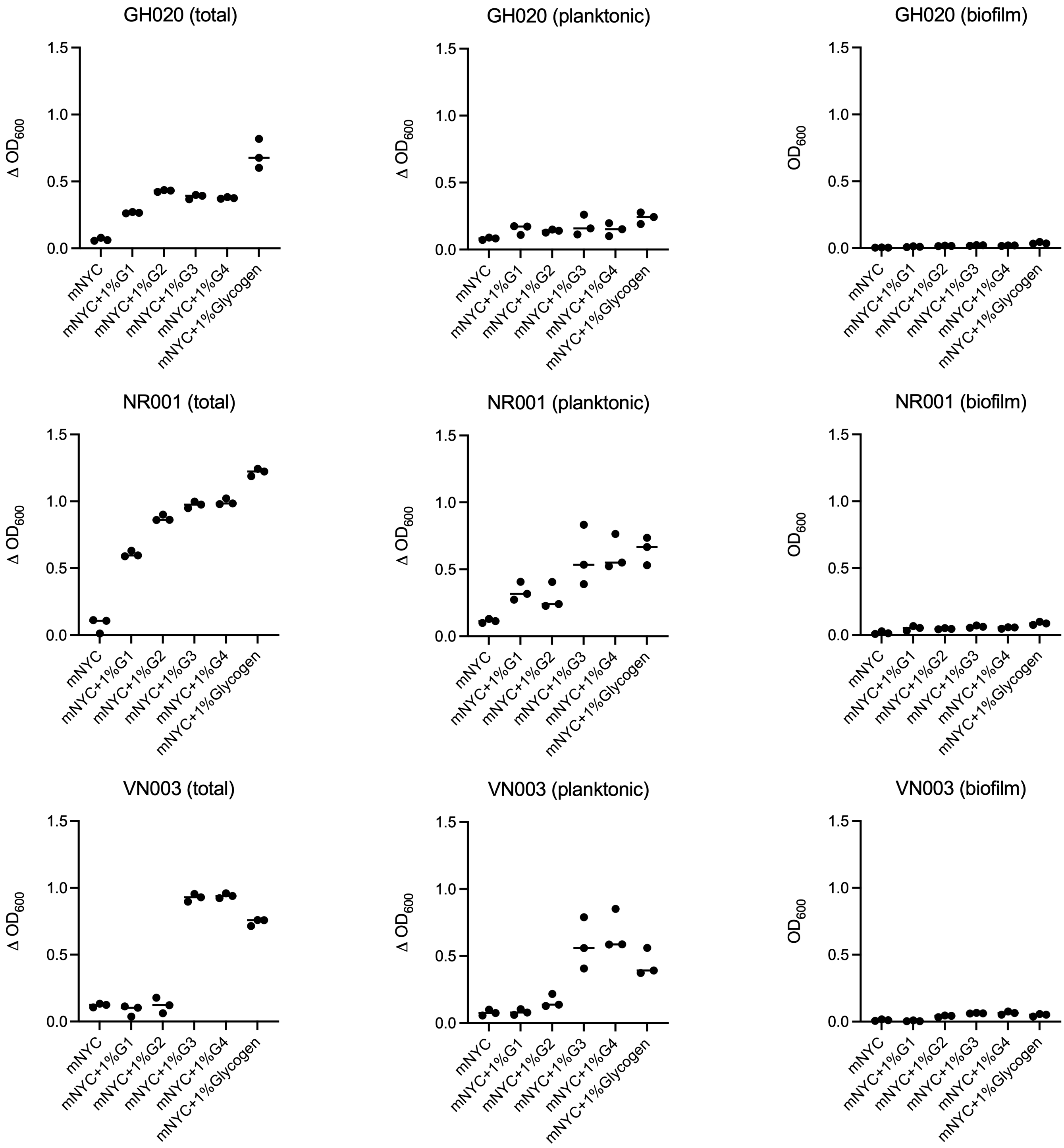

Supplemental Figure 2: Measurement of total (left), planktonic (middle) and biofilm (right) growth for selected isolates of *G. piotii* (GH020), *G. vaginalis* (NR001) and *G. leopoldii* (VN003). Data from three technical replicates are shown; horizontal line indicates the median. Total and planktonic growth are shown as the increase in OD_{600} from 0-48h. Biofilm growth was measured using a crystal violet stain as described in the text.
